# How people with type 2 diabetes in Kuwait manage the condition: A thematic analysis and conceptual framework

**DOI:** 10.1371/journal.pone.0324247

**Published:** 2025-05-27

**Authors:** Zainab Meer, Ebaa Al-Ozairi, Sruthi Ranganathan, Genevie Fernandes, Jay Patel

**Affiliations:** 1 Leeds Beckett University, Leeds, United Kingdom; 2 Clinical Research Unit, Dasman Diabetes Institute, Dasman, Kuwait; 3 School of Clinical Medicine, University of Cambridge, Cambridge, United Kingdom; 4 Centre for Population Health Sciences, Usher Institute, University of Edinburgh, Edinburgh, United Kingdom; 5 Faculty of Medicine and Health, University of Leeds, Leeds, United Kingdom; Public Library of Science, UNITED KINGDOM OF GREAT BRITAIN AND NORTHERN IRELAND

## Abstract

**Background:**

Type 2 diabetes is a growing non-communicable disease burden across the Eastern Mediterranean region, particularly in Kuwait. The methods that patients adopt to manage the condition following diagnosis is poorly understood. This study aimed to explore these methods using a qualitative approach, and develop a conceptual framework characterising the phases that patients transition through.

**Methods:**

This was a qualitative, thematic evaluation of a grounded theory methodology investigating the methods that patients with type 2 diabetes employ to manage their condition. Qualitative coding of semi-structured interview transcripts with 22 patients, over three phases: initial, focused and theoretical, enabled categorical themes and phases to be identified. The findings were synthesised into a conceptual framework that represented the transitional journey.

**Results:**

The development of conceptual framework revealed five transitional categorical phases that characterised the journey: (1) experiencing unusual symptoms; (2) accepting the diagnosis; (3) adopting management strategies; (4) adherence and relapse; and (5) adaptation.

**Conclusion:**

In Kuwait, patients with type 2 diabetes appear to transition through relatively predictable stages in learning to manage their condition. Clinical consideration of this transition could improve the quality of diabetes care provision for these patients.

## Background

Type 2 diabetes is a leading chronic health issue globally [1]. The health implications of diabetes mellitus are serious, and if left untreated or poorly-controlled, the disease can lead to organ failure and death [2]. In addition to the physiological effects of diabetes, the chronic condition also has a profound psychological impact on patients, which is less frequently studied [3].

The International Diabetes Federation [4] found that the health expenditure of diabetes globally amounted $966 billion, and is forecasted to exceed $1 trillion by 2045. A global systematic analysis of diabetes epidemiology in 2021 found that Kuwait represented one of 11 countries in north Africa and the Middle East with a age-standardised diabetes prevalence exceeding 10%, at 15.2% (95% uncertainty interval 14.1–16.3%) [5]. The prevalence of diabetes in the Middle East continues to rise dramatically. The rate of diabetes prevalence in Kuwait represents one of the highest national health metrics globally, and after ischaemic heart disease, the condition is the highest national contributor to deaths and disability.

Although diabetes is incurable and requires careful control, the role of health support from appropriately trained healthcare professionals should be thoroughly explored to minimise the impact of the condition, and complications of the disease. Furthermore, the educational journey of patients in learning to live with diabetes, and adapting to meet the demands of the condition, require qualitative investigation to enable targeted support interventions that maximise the chances of diabetic patients achieving optimal control.

Qualitative studies pertaining to the personal experiences of people living with diabetes, enable a detailed exploration of the psychological and emotive transitions of patients. This study aimed to investigate the categorical phases of transitions that patients with type 2 diabetes patients in Kuwait undergo in the management of the disease. This study also aimed to develop a conceptual framework that represented the methods people living with diabetes use to seek health information to help with the self-management of their condition. The framework attempted to present the breadth and complexities of the various ways adopted by individuals to achieve this.

## Methods

### Overview

This study was a thematic evaluation of a grounded theory methodology. This study also involved the development of a conceptual framework through qualitative coding. The grounded theory methodology applied is described in detail elsewhere [6]. Briefly, 22 adult participants, diagnosed with type 2 diabetes, were recruited across primary, secondary and tertiary care settings across Kuwait. The participants were enrolled across three rounds of recorded, semi-structured interviews both online and in-person, with questions developed through an initial systematic literature review. The transcribed recordings were subsequently coded to identify themes in the methods used by the participants to effectively live with type 2 diabetes.

Hence, this method enabled a far-reaching, exploratory consideration of themes that may be relevant to research question. The research team expected many complexities in the themes across different participants, therefore attempting to synthesise the findings thematically into a conceptual theoretical framework using grounded theory facilitated a constructive process of incorporating the many ways in which patients learn to live with diabetes mellitus.

### Qualitative coding

As grounded theory is an iterative process, both the data collection and data analysis phases were conducted simultaneously. Qualitative coding occurred in three parts: (1) initial coding; (2) focused coding; and (3) theoretical coding.

During initial coding, researchers are required to have a close reading of the data and “study fragments of data – words, lines, segments, and incidents – closely for their analytic import” [7]. Hence, each interview transcript was reviewed by ZM in the native language that the interview was recorded in (i.e., Arabic or English). Coding in the native language of the participant enhances the reliability of data analysis [8,9]. The researcher coded with a view to maintaining open-ended possibilities for different theoretical directions, to facilitate the emergence of novel ideas [7]. Key observations and implicit references were identified using a line-by-line coding technique, and initial codes were developed from these findings.

The focused coding phase followed, consisting of a more direct, and conceptual approach to coding [10]. This phase was reliant upon the preceding phase, where connections were establishing between initial codes, guising a strong analytical direction. The researcher (ZM) extracted the most significant and frequent initial codes to characterise the data. This enabled cross-comparison between the observations of different participants. Codes were developed and refined through this process, initially using NVivo (version 12), followed by Microsoft Excel (16.0).

The final phase involved a more sophisticated process, theoretical coding, where the research team specified potential relationships between categories that had been identified during the focused coding phase. Therefore, this stage captured how the substantive codes related to each other, enabling various hypotheses to integrate into a theoretical model [10,11]. The research team triangulated these findings with the literature.

### Development of a conceptual framework

A conceptual framework representing the substantive theory was developed iteratively, primarily using the themes from the coding. The development of this framework involved applying three further methodological steps prior to synthesising the results into a model. Memo writing was conducted, where notes were recorded outlining the relationships between themes. Constant comparative analysis––considered an essential part of grounded theory method studies–was performed, where data was continually and temporally compared to ensure relevance and consistency in its evaluation [12]. Finally, findings were validated against the preliminary systematic literature review, to guide the development of themes.

### Ethical approval and confidentiality

All participant names were anonymously recorded. Detailed information concerning the nature of the interviews (in terms of setting, recording, transcription, data analysis and anonymity) was provided. All participants voluntarily provided verbal and written consent for insulin in the study. Ethical approval was obtained from the Kuwait Ministry of Health and the Leeds Beckett Research Ethics Committee.

## Results

Coding the interview transcriptions yielded several central themes that defined the experiences of the participants in self-managing type 2 diabetes. We found that participants transitioned through five phases over time: (1) experiencing unusual symptoms; (2) accepting the diagnosis; (3) adopting management strategies; (4) adherence and relapse; and (5) adaptation. However, their transition through these stages was neither guaranteed nor linear. Some participants did not progress to a further stage, some advanced but through different orders, and some returned to a previous stage, when the disease was exacerbated. The full list of categories and codes is shown in Table 1. Participants’ identities have been protected by replacing their names with letters and numbers. The letter at the beginning represents the stage at which the participant was interviewed, with A representing the first stage (family interviews), B representing the second (patients and HCPs) and C representing the final stage where some participants were re-interviewed. The number in all cases signify the order in which the initial interview was conducted.

**Table 1:** List of categories and codes across the five transitional phases.

Phase 1
Experiencing unusual symptoms
Experiencing symptoms of discomfort
Experiencing organ complications
Phase 2
Accepting the diagnosis
Feelings and reactions to the diagnosis
Perceptions of diabetes
Accepting the condition
Changing self-identity
Reconstructing lifestyle
Phase 3
Adopting management strategies
Spiritual coping strategies
Finding positive support
Avoidance
Confronting and seeking solutions
Distraction
Assisting and hindering self-care
Phase 4
Adherence and relapse
Boredom
Surrendering
Phase 5
Adaptation
Fitting self-management into daily life
Adjusting to a new situation
Integrating with the disease
Balancing
Co-existence

### Category 1: *Experiencing unusual symptoms*

This category was constructed from two focused codes: experiencing symptoms of discomfort and experiencing organ complications.

Most participants patients expressed unusual symptoms and organ complications such as, abnormal thirst, frequent urination, blurred vision, heart disease and kidney failure. These two categories are explained in the following sections.

#### Experiencing symptoms of discomfort.

Participants expressed their physiological discomfort from a broad range of symptoms, namely thirst, frequent urination, abnormal sweating, lethargy and fatigue. For instance:

*“I was feeling tired, lazy, dizzy and thirsty all the time…I did not feel comfortable, I felt there was something wrong, but I was not sure if it was signs of diabetes.”* (A: 7)

Although the vast majority of participants had family history of type 2 diabetes, a finding commensurate with the literature, most were largely unaware of the symptoms and their relevance to the condition [13,14]. Diabetes appeared to be a common differential, on the basis of their family history, but the symptoms were frequently attributed to another cause. For example,

*“I thought they were normal symptoms that may happen to anyone. I did not know they are symptoms of diabetes”* (B: 6)

#### Experiencing organ complications.

Some participants suffered organ impairment and dysfunction as either a microvascular or macrovascular complication of diabetes [2]. For example,

*“I had late stage of renal failure due to diabetes.”* (A: 2)

Some of the participants suffered complications such as cardiovascular disease, renal failure and visual problems without an awareness of their connection to type 2 diabetes.

*“I did not know that I have diabetes till I discover that I have kidney failure and vision impairment.”* (B: 9)

Following the onset of symptoms and their progression, participants began seeking information and medical advice to explain the reasoning; this was commonly aimed towards families and friends. Others consulted medical services, used routine medical testing, and public awareness campaigns pertaining to diabetes and glycemic control. The time between the patients’ first suspecting abnormal symptoms, to contacting a healthcare progressional varied widely, from a few weeks to several years.

*“There was a diabetes awareness campaign arranged in one of the malls. I did the test there and discovered that my glucose level was high. I did not believe it in the beginning, and it took me a while to decide to visit a doctor.”* (B: 4)

### Category 2: *Accepting the diagnosis*

Once a diagnosis had been established, participants underwent a phase of accepting the diagnosis, and coming to terms with the condition. This category considered the patients’ feelings and reaction to the diagnosis, their perception of diabetes, accepting the disease, changing self-identity, and reconstructing their lifestyle.

#### Feelings and reactions to the diagnosis.

The majority of participants could accurately recall the moment of their diagnosis, and associated this with powerful reactions from denial, anger, shock, and depression. Some of these emotions influenced adherence to pharmacological management:

*“Since that time I have not taken any medicine. Only after 10 years, I started to take medicine.”* (A: 2)

#### Perceptions of diabetes.

Participants’ views about their state of health were foundational to the narratives they constructed about their condition. These narratives affected the diagnostic acceptance and their approach to management. Some were relieved that the diagnosis was only diabetes, as it excluded more life-threatening diseases. However, these participants appeared to diminish the health implications of diabetes, given its prevalence within their families, and across Kuwait. This comparative weighing of the relative burden of chronic conditions was illustrated as follows:

*“Having diabetes is better than having other more serious diseases like cancer”* (A: 4)

Conversely, the diagnosis also raised concern for many.

*“Diabetes is a nightmare for me. It is even worse than cancer. Once you have diabetes, it starts slowly damaging your arteries, veins and muscles and then attacks all organs”* (A: 2)

#### Accepting the condition.

Most patients eventually accept the condition, identified diabetes as a long-term chronic condition and perceived it as an inevitability. Many relied on religious beliefs to help with their acceptance of the condition, attributing its presence to fate and God.

“*Illness is something destined by God, so I must accept it.”* (B: 5)

There was a reconciliation for some patients transitioning from resentment to acceptance, thoroughly embracing self-management.

*“Now, I have begun to adhere to a healthy diet and I believe that diabetes is a chronic illness that won’t fade away. Therefore, I should prevent more complications through adherence to a healthy diet and taking medications on time. I am keen to know things that can prevent.”* (B: 4)

#### Changing self-identity.

Some participants attributed their diagnosis towards forging a new identity or changing their self-identity. For example, one participant expressed feeling different from others as a result of their condition:

*“When I was initially diagnosed with diabetes, it was an obstacle in my life, especially when I have to travel. Nobody can afford traveling with a person who is diabetic because of the many times I have to go to the toilet. I feel I am not a normal person anymore – I am different from others. So, I have to consider all these things and take cautions when I travel with anyone. I had to learn what things I have to do when I travel as a diabetic person.”* (B: 11)

A shift in identity from an independent person to someone dependent on others was noted for. Some described their struggle with their new identity and recognised the need for adaption to their new lifestyle necessary to manage the disease. For instance,

“*The new situation is very difficult for me, you cannot consume calories as a normal person would. I must calculate the carbs for everything I eat.*” (B: 15)

#### Reconstructing lifestyle.

After accepting the diagnosis, many proactively constructed plans and began making decisions for an enhanced lifestyle, including timely pharmacological adherence, regular glycaemic monitoring, purchasing glucose monitoring devices, calculating nutritional intake, and engaging in exercise.

*“Once I woke up to my new reality, I decided to prevent more complications through adherence to a healthy diet and taking medications on time.”* (B: 4)

*“After I had been diagnosed with a diabetes, I convinced myself that I have to stick to a strict diet.”* (A: 1)

### Category 3: *Adopting management strategies*

This category consisted of six focused codes: spiritual coping strategies, finding positive support, avoidance, confronting and seeking solutions, distraction, and assisting and hindering self-care.

#### Spiritual coping strategies.

As seen in the acceptance phase, religious and spiritual beliefs were identified as important factors in navigating participants through their transitional journey. Religious beliefs and rituals were used as informational sources to assist with the adoption of management strategies:

*“I don’t know how I can describe my life to you. It is a miserable life, but we have to be thankful to God for everything He gives. I always pray and have only faith in God, this is how I am surviving.”* (A: 2)

Religion was also a source of justification and rationale, explaining the disease as an inevitability destined by God:

*“I also understood my situation and realised that it is a plague from God. Illness is something destined by God, so I must accept it.”* (B: 5)

#### Finding positive support.

Support from family, friends and professionals was noted for all participants. The nature of support seeking practices varied, from relatively functional requests (e.g., purchasing equipment to enable optimal glycemic control) to seeking emotional support. The analysis found that the social role of family and friends was critical in diabetic care, enabling assistance early in the disease course, for instance:

*“If I feel that I am upset, I try to go outside with family and friends and have fun and avoid anything that makes me upset. I learned how to overcome and manage my disease by gathering with other people and talking to them.”* (A: 3)

Where family and friends were enlisted for support, patients with diabetes felt it was important for them to bring forward a positive outlook. Patients actively avoided people with negative influences

*“People who have diabetes should not talk with other people who are negative but talk with positive people; negative people could depress them even more. I try to sit with people who are funny and make my life more interesting and fun and who can give me hope in my life.”* (A: 3)

#### Avoidance.

The majority of patients used a constructive mindset to confront the condition, however some used avoidance as a tool to disengage with, or evade the need to manage the disease. This was either behavioural avoidance (i.e., failing to test blood glucose regularly or not adhering to a nutritional care plan) or cognitive avoidance (i.e., avoiding information). There was a meaningful correlation between coping through avoidance and the desire for co-existing with the condition peacefully. For example, one participant used behavioural avoidance to avoid further worry:

*“I have more than one glucose testing meters in my house and my daughter keeps buying me the test stripes, but I would never use them. I don’t like to use them. Because if I discover that my blood sugar level is high, I would start to become tense and worried.”* (A: 4)

Avoiding information related to diabetes was an example of cognitive avoidance exercised by some participants as they found that such information increased their stress levels. One participant explained that when reading about her sister’s illness, she became overwhelmed and depressed:

*“I do not like to search for information. This is because I had a bad experience when my sister was diagnosed with blood cancer and I started searching and reading about the disease. I became depressed and kind of frustrated with the information I read. I felt that she would not be able to recover from the disease. The information was embedded in my mind and gave me an impression that the cancer would spread quickly in her body and she will die…We kept reading as we were uncertain and worried, but it made our lives difficult. That’s why, when I was diagnosed with diabetes, I did not seek information from the internet or by searching through Google; I know that the more I dig into reading about it, the more I will be overwhelmed and depressed. I like things to be kept hidden from me, so I can live peacefully.”* (B: 10)

#### Confronting and seeking solutions.

Soon after their diagnosis, patients sought strategies to confront and mange the condition, through adherence to management strategies and optimising disease control. For example,

“*I try to take my meals in a timely manner and I do not delay it as much as possible. I also try to reduce the carbohydrates in meals*.” (B: 5)

Almost all participants mentioned their commitment to self-care management. Comparative analysis showed that some participants relied on biomedical management before switching to, or combining with traditional medicine approaches. However, patients soon acquired a strong opinion on whether those approaches were likely to yield dividends for their diabetes control. The rationale for searching for, and the subsequent use of alternative medicines was the hope to be cured as noted by healthcare providers. The behaviours around traditional medicines are illustrated below:

*“I take some Murah (a particular herb), which I always keep with me in order to reduce high sugar levels.”* (B: 11)

*“I tried taking Cinnamon but to no effect!”* (B: 14)

*“We constantly reflect on why patients seek medication through herbs and alternative treatments? It seems that patients do this in the hope that it will cure them from diabetes.”* (B: 19).

#### Distraction.

While some attempted to confront the condition constructively, others applied distraction techniques by engaging in alternative activities, or completely avoiding situations that may incur stress (e.g., engaging with hobbies, gathering with others, and not worrying about the condition). Some participants stated that this provided relief and prevented their blood glucose levels elevating:

*“What I do is just rest and try not to think or get worried about it because, if I start thinking, my blood sugar will rise higher.”* (A: 4)

Others explained that they aim to avoiding thinking and worrying about the condition excessively. This helped control their diabetes, adapt to life with it, and co-exist with the disease:

*“I try as much as possible not to feel concerned about it so I can coexist with it and to stop it from taking hold of me…because I do not take diabetes seriously, it helped me adapt and coexist with the disease.”* (B: 8)

#### Assisting and hindering self-care.

This focused code was constructed when the participants shared experiences of the role of different factors on the degree of assistance and hindrance on patients’ self-care management, including social and culture, environmental, management and clinical factors.

Family and social support was important in assisting patients’ self-care management from the provision of information, food recipes, and assistance in the use of technology (e.g., medicine alarms applications).

*“I am illiterate. I don’t know how to read. However, I get the information I need from my children and other people around me. My children are educated. My daughter is a teacher, the other daughter is a secretary and my son is an engineer”* (B: 5)

### Category 4: *Adherence and relapse*

The fourth category was constructed from two parts: developing boredom and surrendering.

#### Boredom.

This category developed when the participants shared their experiences of becoming bored over time with their daily self-care management routine. For example,

*“I do not follow up my appointments regularly. The point is that this matter has become a boring routine; tedious and unchangeable. Even the follow-ups at the clinic are the same and nothing new happens except the dispensing of medicine. Nobody tells you anything in the clinic.”* (A: 4)

The significance of boredom as a transitional phase was that some decided not to commit to management strategies as stringently, particularly adherence to nutritional programme and medication, and returned to previous habits, with waning interest in learning about their disease. Consequently, these patients relapsed:

“I haven’t been okay recently since I was uncommitted to the medication due to my life tensions and pressures. I felt that people around me did not accept the idea that I was sick. I didn’t feel like taking the diabetes medicine and I was stubborn about taking the medicine. I don’t know why it has become a tiring course of treatment and I feel as if it is useless.” (B: 8)

Healthcare professionals were also able to verify this as a hallmark feature of the diabetes self-care journey:

*“At the beginning, especially in those patients who recently got diabetes, they demonstrate enthusiastic behaviour which is reflected in their regular monitoring their glucose levels. However, after that, it turns into boring routine for them, then their enthusiasm gradually fades and they become careless, particularly when they perceive that their disease is chronic and inherent. Most of the careless patients are those who are diabetic for more than five years.”* (B: 17)

#### Surrendering.

Surrendering was mentioned in the context of protracted negative experiences with diabetes, such as feeling controlled by the disease, one even described it as living with an enemy:

*“It is diabetes that controls me – not the opposite. It is like living with your enemy, even if you hate it you have to live with it peacefully.”* (A4)

Some participants surrendered to the disease once they believed they had exercised most avenues to manage their illness could not find ways to make themselves feel better.

### Category 5: *Adaptation*

The final category was attributable to an adaptive phase. Adaptation consisted of fitting self-management into daily life, adjusting to a new situation, integrating with the disease, balancing, and culminating with co-existence.

#### Fitting self-management into daily life.

This focused code harnessed the participants’ narratives around fitting and adapting self-care management into their lifestyle. Once a person has been diagnosed with diabetes, their life goes on, and they attempt to ‘fit’ their daily self-care management routine into their lives. Many devised novel methods to adapt to the new lifestyle, for example:

*“I try as much as possible to adapt the daily routine to my daily life…I have become more punctual as I set the alarm to the injection schedules so that I should not forget it. I tried to adapt myself to my disease…Since I was diagnosed with type 2 diabetes, I felt scared and I started to walk half an hour every morning – as well as in the afternoon. In the morning time I make it as part of my daily routine as I walk to work from the parking area to the workplace.”* (B: 5)

#### Adjusting to a new situation.

This reflective code was mentioned by participants while sharing thoughts and feeling about their adjustments to effectively adapt to a new biomedical situation (i.e., avoiding gatherings, reducing dietary fats, and ensuring that the timing of activities did not disrupt their medication and nutritional regimen). Frequently, a long period of trying different strategies and seeking various sources of information to gain the skills necessary for their self-care management was employed.

*“I used to take care of my mum. This experience helped me to know more about the disease…I don’t like reading. So, I have not read about diabetes, but I prefer taking information from other diabetic patients…I believe that if I seek more information about diabetes I become more worried about the symptoms I have, so I prefer to avoid seeking information about diabetes…I look after myself and my health and control my eating habits…I managed to control my diet by not eating sweets and eating less fruit…I adjusted with the new situation and became my own nurse.”* (B: 14)

#### Integrating with the disease.

In addition to finding time to fit self-management into daily life, the overarching objective was to integrate more coherently with the disease:

*“I have to carry on with life. Diabetes become part of me and I have to learn how to manage it, so I can coexist with it peacefully.”* (B: 9)

When participants accepted that diabetes management required a symbiotic integration with their current lifestyle and routine, they were able live and co-exist with the condition peacefully:

*“I am learning to control and mange my disease. I always wanted to know everything about it. The information I gathered helped me to learn and manage my disease and integrate with the person who I am now. I do not feel worry anymore as I try to read and understand my illness and learn how to live peacefully with it.”* (B: 5)

#### Balancing.

Some participants mentioned strategies that have helped balance their needs with their daily self-care management:

*“My diet has come under the supervision of the dietician. I have become more committed. However, I don’t deprive myself of my favourite food, but take it in moderate amounts.”* (B: 3)

On the other hand, some experienced difficulty in adapting to the self-care management process to live a normal life and balancing their needs. Therefore, regarded themselves as ‘becoming abnormal’ or ‘different from others’. Many participants felt they had lost the joy of everyday life:

*“I feel I am not a normal person anymore – I am different from others. I cannot travel like before; or I have to consider all these things and take cautions when I travel with anyone.”* (B: 2)

#### Co-existence.

This focused code was consistently referred by the participants throughout the interviews. For most of the participants ‘*co-existing’* was a consequence of them accepting the diagnosis, adapting and living with the disease. Co-existing was defined differently among participants, as acceptance, reconciling with the disease, or aiming not to surrender to it. Although some had setbacks to managing their condition due to particular circumstances, such as social engagements or a lack of social support, after time they were able to galvanise themselves and realise their need to progress and work constructively in the self-care process:

*“Coexistence is accepting the fact of being diseased as being destined by God while not surrendering oneself to the disease. For me, coexistence is to learn to accept and live side by side with the disease and build a relationship with it.”* (B: 13)

*“Coexisting means facing the disease and accepting it. I was unwilling to accept it at the beginning for a while that is because I couldn’t imagine how I would take medication on a daily basis.”* (B: 8)

### Constant comparative analysis

Constant comparative analysis was an essential component of data analysis, as this allowed the research team to identify information behaviours that were changing from the time of diagnosis, and subsequently throughout the course of the disease. For example,

*“I had this disease since 1990; it is an old disease. At the beginning I was young and used to take care of myself. Now I don’t bother to learn anything about the disease anymore.”* (A:4)

The dynamic nature of information behaviour change was also corroborated by health care providers, for example:

*“At the beginning, especially in those patients who recently got diabetes, they demonstrate enthusiastic behaviour which is reflected in their regular monitoring of their glucose levels. However, after that, it turns into a boring routine for them, and their enthusiasm gradually fades and they become careless, particularly when they perceive that their disease is chronic and inherent. Most of the careless patients are those who are diabetic for more than five years.”* (B:17)

The research team had understood that family and friends were supportive stakeholders in the acceptance and management of diabetes. However, through comparing interview transcripts, friends and family were often seen as an obstacle towards optimal disease management, illustrated by a participant who was unable to commit to sustained healthy nutrition:

*“I used to follow a very strict diet and I lost weight, but then my friends in dewaneya ruined everything (laughing). They invited me so many times for dinner and I could not refuse as part of our culture and since then I could not commit again to a healthy diet.”* (A: 7)

### Development of a conceptual framework

The final stage of analysis involved the development of theoretical categories constituting the substantive theory of learning to manage and co-exist with diabetes. The phases of data collection characterised a sense of continuum in the information behaviours of patients with diabetes in Kuwait. Some participants attempted to summarise this chronological process, which largely verified the conglomerate of findings across the interviews more broadly. It was also clear that the information behaviour process was helpful in setting expectations for diabetes self-care, and helping to temper anxieties and stress about independent diabetes self-management. Three participants illustrated these broader dimensions:

*“You keep trying learning ways to manage your diabetes. you start learning the way you can cope with idea of having the disease in your life, then you look for information to know how to manage it, then you keep trying by yourself till you find a way to live with it. It just never ends.”* (C: A4)

*“It is a long journey, and I should carry on with life. Diabetes become part of me, and I am learning how to manage it sometimes by seeking and asking my doctor but at times avoiding knowing details as much as I can so I can coexist with it peacefully.”* (C: B8)

*“I keep learning and educating myself to manage my disease. I wouldn’t know how to control my sugar level if I don’t have the information needed. I also learned how to cope well and avoid stress from information I have from my doctor and sometimes from reading things from the internet. This helped me a lot in learning how to live with it in peaceful mind.”* (C.B3)

**Fig 1:**
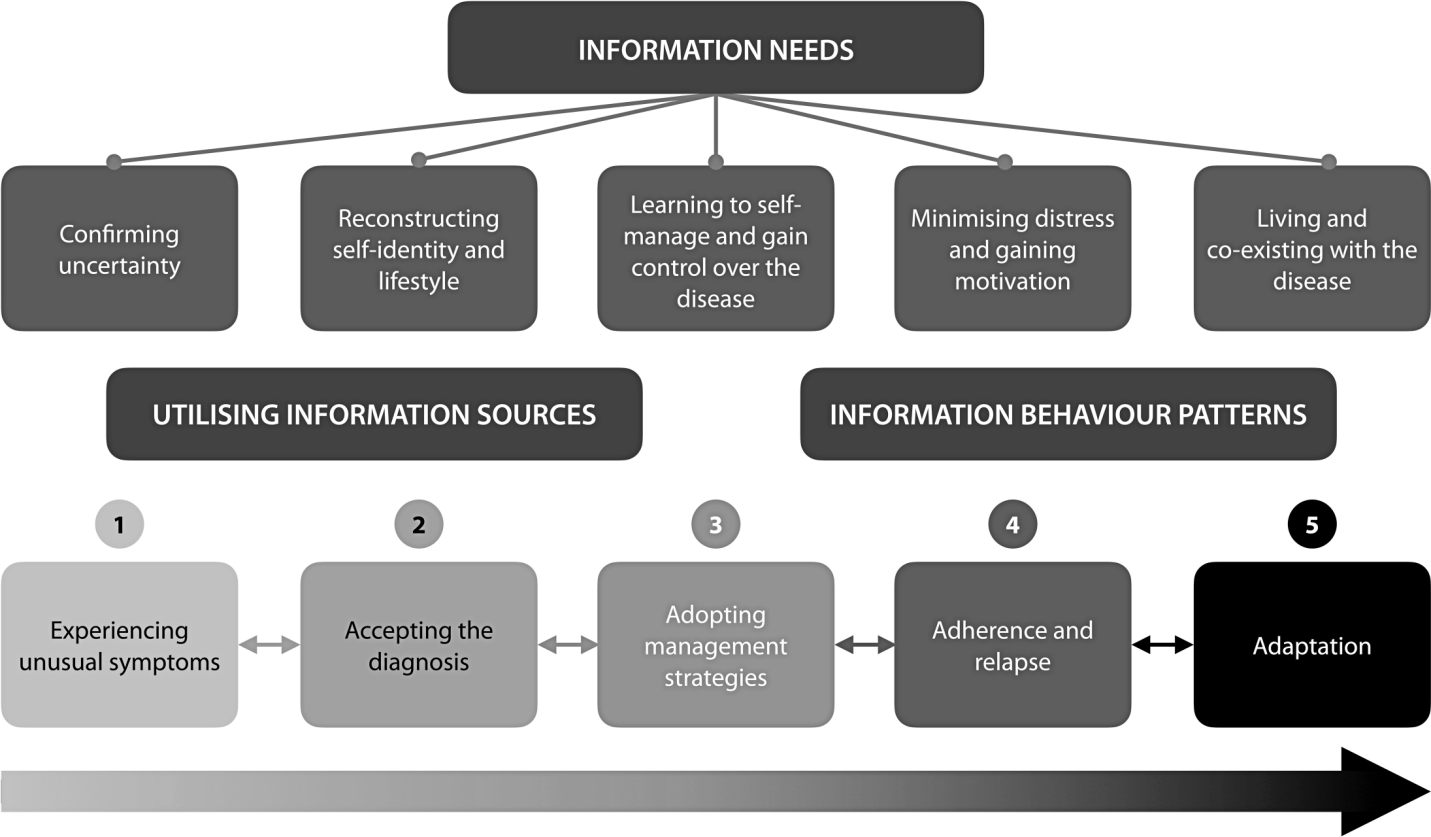
A conceptual framework and substantive theory for learning to self-manage and co-exist with type 2 diabetes.

The conceptual framework encompassed the broad concepts and more nuanced complexities for a substantive theory model of learning to manage and co-exist with diabetes is summarised (Fig 1), reinforcing the value of a grounded theory methodology in enabling this result. The model connects three aspects of information behaviour: (1) information needs; (2) the utilisation of information sources; and (3) information behaviour patterns.

## Discussion

This thematic evaluation of a grounded theory study characterised the categorical phases that patients with type 2 diabetes transition through in learning to live with the disease. Findings from the study showed that participants attributed particular meanings to their experience and continually re-interpreted this throughout the illness trajectory. The process of learning to self-manage and co-exist with diabetes was described as a continuous learning process in order to live and co-exist with the disease. A dynamic, transitional process through five key phases over the time period (experiencing unusual symptoms; accepting the diagnosis; adopting management strategies; adherence and relapse; and adaptation) was a pattern of similarity, whilst simultaneously demonstrating the variability in strategic approaches across each stage.

A dynamic, transitional process through five key phases over the time period (experiencing unusual symptoms; accepting the diagnosis; adopting management strategies; adherence and relapse; and adaptation) was a pattern of similarity, whilst simultaneously demonstrating the variability in strategic approaches across each stage. This learning process was not necessarily characterised by a seamless developmental journey from beginning to end, but more accurately represented more dynamic, disorderly process, with repetition and recapitulation, of information they receive, seek or avoid to optimal manage their condition.

An important aspect of developing a framework that accurately reflected participants’ experiences, was to account for the backwards motion between phases, in the context of the overall transition from the first to the fifth phase (Figure 1). For example, many participants, who experienced unusual symptoms delayed seeking information and medical advice thinking that their symptoms were normal and hoping that their symptoms would disappear. This delayed transitioning as swiftly to the *accepting the diagnosis* phase. Only a few participants transitioned from adopting management strategies to adapting without suffering any relapse. Likewise, some participants adapted to their self-care management but relapsed due to four key factors: social responsibility; burnout; disinterest or boredom with their routine; or work commitments. Also, participants regressed from the *adaptation* phase to *adopting management strategies.* This regression was typically triggered by experiencing new symptoms or complication.

Findings from the comparative analysis encouraged the research team to re-evaluate the investigation of information behaviour across different time periods following diagnosis and considering how the temporal dimension influenced the participants ‘information behaviour’. As the burden of type 2 diabetes continues to grown in Kuwait, and across the Eastern Mediterranean, enhanced awareness of the lived experience of type 2 diabetes patients and their information behaviours.

### Limitations

There are limitations to address in this study. Despite the value of a thematic evaluation using the grounded theory methodology as a baseline, this limits the range of data interpretation, as the research team were ultimately required to synthesise themes into categorial codes. To a degree, this limitation was reduced by presenting many sub-codes detailed throughout the manuscript. Additionally, the findings presented from this study are inherently dependent upon the reliability of qualitative coding. Although a hierarchical approach to coding was applied, enabling continual consolidation of codes, the research team may have missed critical codes across the transcripts.

The conceptual framework may represent a simplification of a more complex model, or may not comprehensively capture additional phases or transitional directions, that may have arisen due to a small sample size (from the initial grounded theory study) or through the research team failing to identify these from the transcripts.

## Conclusion

Living with type 2 diabetes involved many changes for patients, namely emotional developments, reconstruction of lifestyles and identities, and changes in the methods used to locate and use information. This study highlighted the dynamic process of learning to manage and co-exist with type 2 diabetes for patients in Kuwait, as a core component of disease management. The development of a conceptual framework demonstrates how the provision of information could be optimised for people with the condition. Patients with type 2 diabetes appeared to transition dynamically through five phases: experiencing unusual symptoms; accepting the diagnosis; adopting management strategies; adherence and relapse; and adaptation, in a way that was sensitive to situational changes. Further research is needed to develop similar frameworks across type 2 diabetic patients in different sociodemographic contexts, to advance the agenda on global diabetes control.
